# Phasic firing of dopaminergic neurons in the ventral tegmental area triggers peripheral immune responses

**DOI:** 10.1038/s41598-022-05306-8

**Published:** 2022-01-27

**Authors:** Tasuku Kayama, Yuji Ikegaya, Takuya Sasaki

**Affiliations:** 1 Graduate School of Pharmaceutical Sciences, The University of Tokyo, Tokyo, 113-0033 Japan; 2 Department of Pharmacology, Graduate School of Pharmaceutical Sciences, Tohoku University, 6-3 Aramaki-Aoba, Aoba-Ku, Sendai, 980-8578 Japan; 3 Institute for AI and Beyond, The University of Tokyo, Tokyo, 113-0033 Japan; 4 Center for Information and Neural Networks, National Institute of Information and Communications Technology, Suita City, Osaka, 565-0871 Japan

**Keywords:** Emotion, Reward, Neuroscience

## Abstract

Dopaminergic neurons in the ventral tegmental area (VTA) play a crucial role in the processing of reward-related information. Recent studies with pharmacological manipulations of VTA neuronal activity demonstrated a VTA-induced immunoenhancement in peripheral organs. Here, to examine the detailed physiological dynamics, we took an optogenetic approach in which VTA dopaminergic neurons were selectively activated with millisecond precision. Optogenetic phasic, rather than tonic, stimulation of VTA dopaminergic neurons increased serum cytokine levels, such as IL-2, IL-4 and TNF-α. These results provide direct evidence to link dopaminergic neuronal phasic firing to peripheral immunity. Next, we tested whether cytokine induction in male mice was boosted by female encounters, a natural condition that induces increased active VTA neurons and gamma power. Female encounters increased serum IL-2 levels, which were abolished by pharmacological inhibition of VTA neuronal activity. Taken together, our results highlight the importance of the brain reward system in the treatment and management of immune-related disorders.

## Introduction

Animals develop positive feelings and behavior when they anticipate or receive rewarding stimuli. Several pieces of recent evidence support that brain activity related to reward-seeking behavior, experiences, and motivation has beneficial effects on not only psychiatric and emotional states but also widespread immune responses, the so-called neural–immune interactions, via activation of the hypothalamic–pituitary–adrenal (HPA) axis and the peripheral nervous system^1,2^. A key neuronal network that mediates positive emotions, expectations, and reinforcement is the dopaminergic neurons in the ventral tegmental area (VTA), which provides a primary source of dopamine (DA) in the mesolimbic reward system^3–5^. Neuroimaging studies from humans have demonstrated strong activation of the VTA by sexual behavior^6^ and substantial release of endogenous dopamine by placebo effects^7^. At the neuronal level, a series of studies from rodents demonstrated that dopamine neurons in the VTA are activated by reward prediction errors^8^, sexual appetitive reactions^9,10^, and oral sucrose stimulation^11^. In addition to these neurophysiological measurements, several recent studies have supported the insights of VTA-induced immunoenhancement by showing that experimentally activating the mesolimbic reward system increased natural killer (NK) cell cytotoxicity^12^ and boosted antibacterial and antitumor immune responses^13,14^.

While these studies began to highlight the central role of the VTA in neuroimmune interactions, several unresolved issues remain to be clarified. In particular, millisecond-level spike patterns of VTA dopaminergic neurons are crucial, as they exhibit two distinct firing modes: (i) low-frequency tonic firing that sustains the resting concentration of dopamine and (ii) high-frequency phasic firing that encodes reward signals and evokes a transient rise in dopamine^15–18^. First, which types of activity patterns in the VTA impact immunity? The chemogenetic stimulation utilized in previous studies does not clarify this idea due to the impossibility of manipulating dopaminergic (DA) neuronal spikes on the millisecond timescale. Second, do VTA-induced immune reactions occur under naturally rewarding conditions? While the early studies described above utilized artificial stimulation, such as electrical and chemogenetic stimulation, it remains to be fully determined whether endogenous activation of the mesolimbic reward system by positive experiences (such as mating and feeding) is sufficient to mobilize immune responses.

To address these issues, we utilized optogenetic tools^19^, which have been shown to separately mimic phasic and tonic VTA dopaminergic neuronal activity patterns and dissect their functional significance^16,18,20^. Next, we tested how the cytokine levels in male mice are affected by a female encounter, as a naturally occurring reward, which has been shown to endogenously activate the mesolimbic system^9,21,22^.

## Results

### Activation of VTA dopaminergic neurons elevates serum cytokine levels

**Figure 1:**
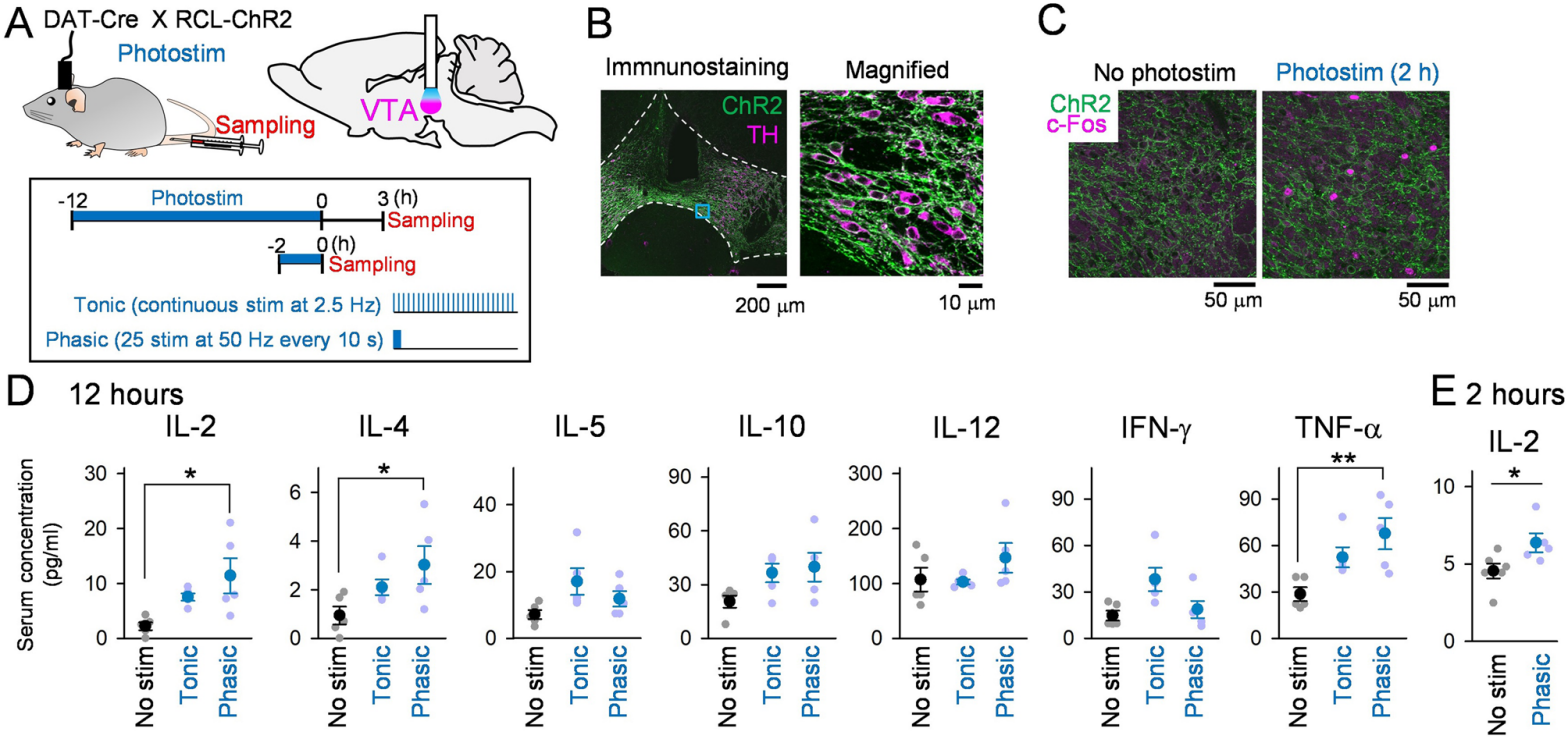
Photostimulation of the VTA DA neurons increases serum cytokine levels. (**A**) In DAT-Cre × RCL-ChR2-eYFP mice, photostimulation was applied to the right VTA through an optic fiber for 12 h or 2 h, and a blood sample was collected from the tail vein after 3 h or immediately, respectively. The bottom inset shows the experimental paradigm. (**B**) (Left) A coronal section showing neurons in the VTA immunostained with ChR2-eYFP (green) and TH (magenta) antibodies. The cyan region is magnified in the right panel. (**C**) Immunostaining of VTA neurons with ChR2-eYFP (green) and c-Fos (magenta) antibodies after 2 h of VTA photostimulation. (**D**) Serum concentrations of seven cytokines tested after 12-h VTA photostimulation (*n* = 5 mice each). Each thin dot represents one mouse. *P < 0.05, **P < 0.01. (**E**) Same as D but serum concentration of IL-2 was tested after 2-h VTA phasic photostimulation (*n* = 6 and 5 mice). *P < 0.05.

VTA neurons are heterogeneous, including dopaminergic and nondopaminergic (mostly GABA) neurons^23–25^. To selectively activate VTA dopaminergic neurons, we obtained bigenic DAT-Cre × RCL-ChR2-eYFP mice (Fig. 1A) by crossing two mouse lines: one in which the expression of Cre is driven by the dopamine transporter (DAT) promoter, a dopaminergic neuron-specific promoter^26^, and the other in which ChR2-eYFP is expressed in a Cre-dependent manner. Here, only male mice were used, unless otherwise specified. We performed immunohistochemical labeling of VTA neurons to confirm the selective expression of ChR2-eYFP. The majority (~ 89%) of the ChR2-eYFP-positive VTA neurons were stained with TH (tyrosine hydroxylase), a specific marker of dopaminergic neurons, demonstrating highly efficacious transduction of TH-expressing cells (Fig. 1B). An optical fiber was implanted into the right VTA in DAT-Cre × RCL-ChR2-eYFP mice, and photostimulation was applied (Fig. 1A). Figure 1C shows typical immunostaining for c-Fos, an immediate-early gene as a marker of neuronal activation, after 2 h of phasic photostimulation. At the region directly beneath the optic fiber, strong c-Fos expression was observed in VTA ChR2-eYFP-positive neurons, confirming the successful activation of VTA dopaminergic neurons.

We next examined whether VTA photostimulation activates peripheral immune responses. It has been shown that phasic VTA DA neuronal activity induces a large transient rise in dopamine release, whereas tonic activity induces low sustained release^18^. Therefore, we utilized one of two photostimulation patterns, mimicking these distinct VTA dopaminergic neuronal firing patterns^18^, either (1) tonic stimulation in which single pulses (~ 30 mV, 15 ms) were continuously applied at 2.5 Hz, or (2) phasic stimulation in which a burst of 25 pulses at 50 Hz were intermittently applied every 10 s^16^ (Fig. 1A, C). This phasic stimulation protocol has been shown to induce VTA neuronal spikes at a success rate of approximately 30%^16^. Both stimulation protocols lasted for 12 h and had the same total number and duration of pulses. Blood samples were collected from the tail veins 3 h after photostimulation (Fig. 1A), and the levels of serum cytokines were quantified. After 12-h phasic photostimulation, serum IL-2, IL-4, and TNF-α levels increased significantly (Fig. 1D; IL-2 : *F* (2, 12) = 5.71, *P* = 0.018, one-way ANOVA. *P* = 0.014, Tukey’s test; IL-4 : *F* (2, 12) = 3.87, *P* = 0.050, one-way ANOVA. *P* = 0.041, Tukey’s test; TNF-α : *F* (2,12) = 7.04, *P* = 0.0090, one-way ANOVA. *P* = 0.008, Tukey’s test), compared with control conditions without photostimulation, while serum IL-5, IL-10, IL-12, and IFN-γ levels were unchanged (Fig. 1D). On the other hand, no significant changes in any of the serum cytokine levels tested were observed after 12-h tonic photostimulation (IL-2 : *P* = 0.17, IL-4 : *P* = 0.30, TNF-α : *P* = 0.099, Tukey’s test after one-way ANOVA) compared with control conditions. While it appears that several cytokine levels from the tonic stimulation group are higher and lower than the control and the phasic stimulation group, respectively, no significant differences in any of the serum cytokine levels were observed between phasic and tonic photostimulation (IL-2 : *P* = 0.36, IL-4 : *P* = 0.46, TNF-α : *P* = 0.35, Tukey’s test after one-way ANOVA), while we need to note that our analyses from relatively small sample size may fail to detect pronounced significance from the tonic stimulation group. These results suggest that a subset of serum cytokines is effectively elevated by phasic, rather than tonic, activation of VTA dopaminergic neurons. Having verified the effect of phasic firing of dopaminergic neurons on immune responses, we tested whether phasic photostimulation in shorter periods (2 h) could induce the similar effect (Fig. 1A, E). We observed a similar significant increase in the serum IL-2 level (Fig. 1E; *t*_10_ = 2.33, *P* = 0.042, Student’s *t*-test), suggesting that phasic activation for 2 h is sufficient to enhance serum IL-2 level.

### A female encounter experience activates the reward circuit

**Figure 2:**
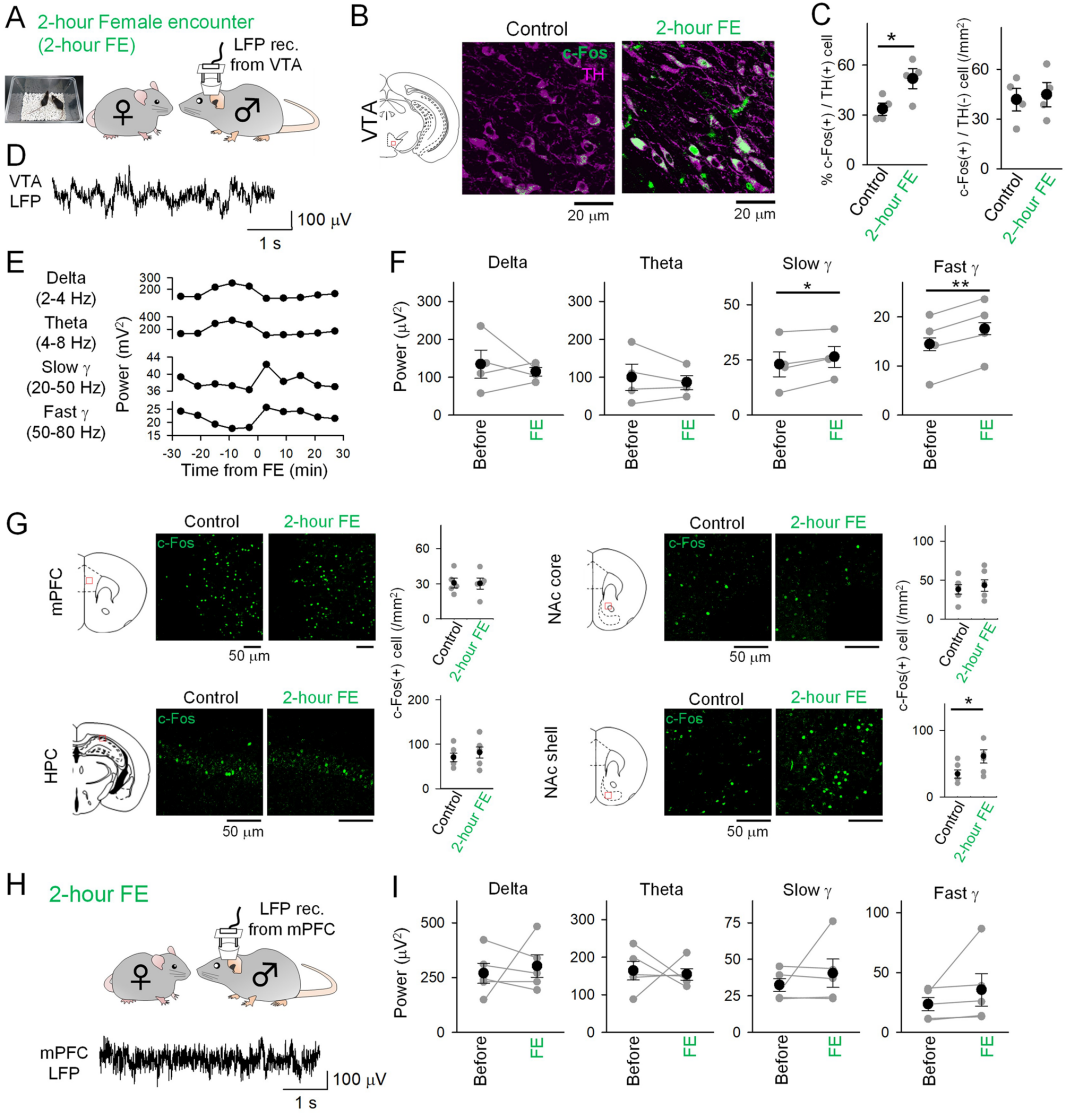
Activation of the VTA neurons in male mice by a female encounter. (**A**) A male mouse was allowed to freely interact with a female mouse for 2 h, termed a female encounter (FE). Some mice were implanted with electrodes in the VTA. (**B**) Representative images of the VTA neurons labeled with anti-c-Fos (green) and TH (magenta) antibodies after the FE. (**C**) (Left) The percentages of both c-Fos-positive and TH-positive neurons among all TH-positive neurons in the VTA (Control, *n* = 4 mice; 2-h FE, *n* = 4 mice) (Right) The percentages of c-Fos-positive and TH-negative neurons among TH-negative neurons in the VTA (Control, *n* = 4 mice; 2-h FE, *n* = 4 mice). *P < 0.05. (**D**) Local field potential (LFP) signals were recorded from the VTA during the FE. (**E**) FE-induced time changes in the VTA LFP power at each frequency band in mice. (**F**) Comparisons of the VTA LFP power between 30-min before (before) and 30-min after (FE) the onset of FE in all mice (*n* = 4 electrodes from 4 mice). Each gray dot represents one mouse. *P < 0.05, **P < 0.01. (**G**) (Left) Immunohistochemical analysis of c-Fos in each brain region in male mice. (Right) Quantification of c-Fos-positive neurons in each brain region (Control, *n* = 5–6 mice; 2-h FE, *n* = 5–6 mice). (**H**) LFP signals were recorded from the mPFC during the FE. (**I**) Similar to E but for mPFC LFP power (*n* = 5 electrodes from 3 mice).

We next asked whether more natural conditions could elicit VTA activation-mediated immune responses. Based on previous observations that dopamine in the reward system is crucial for the expression of male sexual behavior^27^, we utilized a female encounter (FE) paradigm as a reward stimulus for male mice in which a female mouse is placed in the home cage of a male mouse (Fig. 2A). Based on the observation that activation of dopaminergic neurons for 2 h was sufficient to induce the immune response (Fig. 1E), the period of the FE paradigm was set to be 2 h. During the 2-h FE period, all male mice showed precopulatory behavior with motivation to approach and engage in extensive olfactory investigation of the female mice but showed no ejaculation behavior. FE for 2 h induced significant increases in the proportions of c-Fos-positive dopaminergic neurons in the VTA (Fig. 2B, C left; *t*_7_ = 2.52, *P* = 0.040, Student’s *t*-test). On the other hand, such effects were not observed in TH-negative (non-dopaminergic) neurons (Fig. 2C right, *t*_7_ = 0.31, *P* = 0.77). These results suggest that increases in neuronal activity defined by c-Fos expressions are specific to dopaminergic neurons in the VTA. To examine more details of the neuronal activity patterns, LFP signals were recorded from the VTA in freely moving mice that were exposed to a FE (Fig. 2D). LFP signals were continuously monitored from 30 min before to 120 min after the FE. VTA LFP patterns were classified into delta (2–4 Hz), theta (4–8 Hz), slow gamma (20–50 Hz), and fast gamma (50–80 Hz) oscillations. As shown in a representative example in Fig. 2E, A transient change in gamma power was observed after FE. Overall, there were significant changes in LFP power at the slow gamma and fast gamma bands during FE, whereas no changes were observed in delta or theta power (Fig. 2F; delta, *t*_3_ = 0.62, *P* = 0.58; theta, *t*_3_ = 0.826, *P* = 0.47; slow gamma, *t*_3_ = 3.43, *P* = 0.042; fast gamma, *t*_3_ = 12.71, *P* = 0.0010, paired *t*-test). These results suggest that high-frequency neuronal activity in the VTA is specifically altered in response to a FE, which is consistent with the increased activity in the mesolimbic dopamine system of males after female stimulus^21,22,28,29^. DA neurons project to various brain regions, including the medial prefrontal cortex (mPFC), hippocampus (HPC), and nucleus accumbens (NAc), consisting of the core and shell. After 2 h of FE, these brain regions were also stained by c-Fos immunohistochemistry. The proportion of c-Fos-positive neurons increased in the NAc shell (Fig. 2G; *t*_11_ = 2.26, *P* = 0.045, Student’s *t*-test), a region implicated in the reward circuitry. On the other hand, no significant changes in c-Fos expression were detected in the other brain regions (Fig. 2G; mPFC, *t*_9_ = 0.060, *P* = 0.95; HPC, *t*_11_ = 0.71, *P* = 0.49; NAc core, *t*_11_ = 0.53, *P* = 0.61; Student’s *t*-test). In addition, we found no prominent changes in LFP power in the mPFC at any frequency band tested (Fig. 2H, I; delta, *t*_4_ = 0.43, *P* = 0.69; theta, *t*_4_ = 0.27, *P* = 0.80; slow gamma, *t*_4_ = 0.88, *P* = 0.428; fast gamma, *t*_4_ = 1.203, *P* = 0.295, paired *t*-test). Taken together, these results again confirm that the reward circuit, including the VTA and NAc shell, is activated during our FE protocol.

### A female encounter experience elevates serum cytokine levels through the VTA

**Figure 3:**
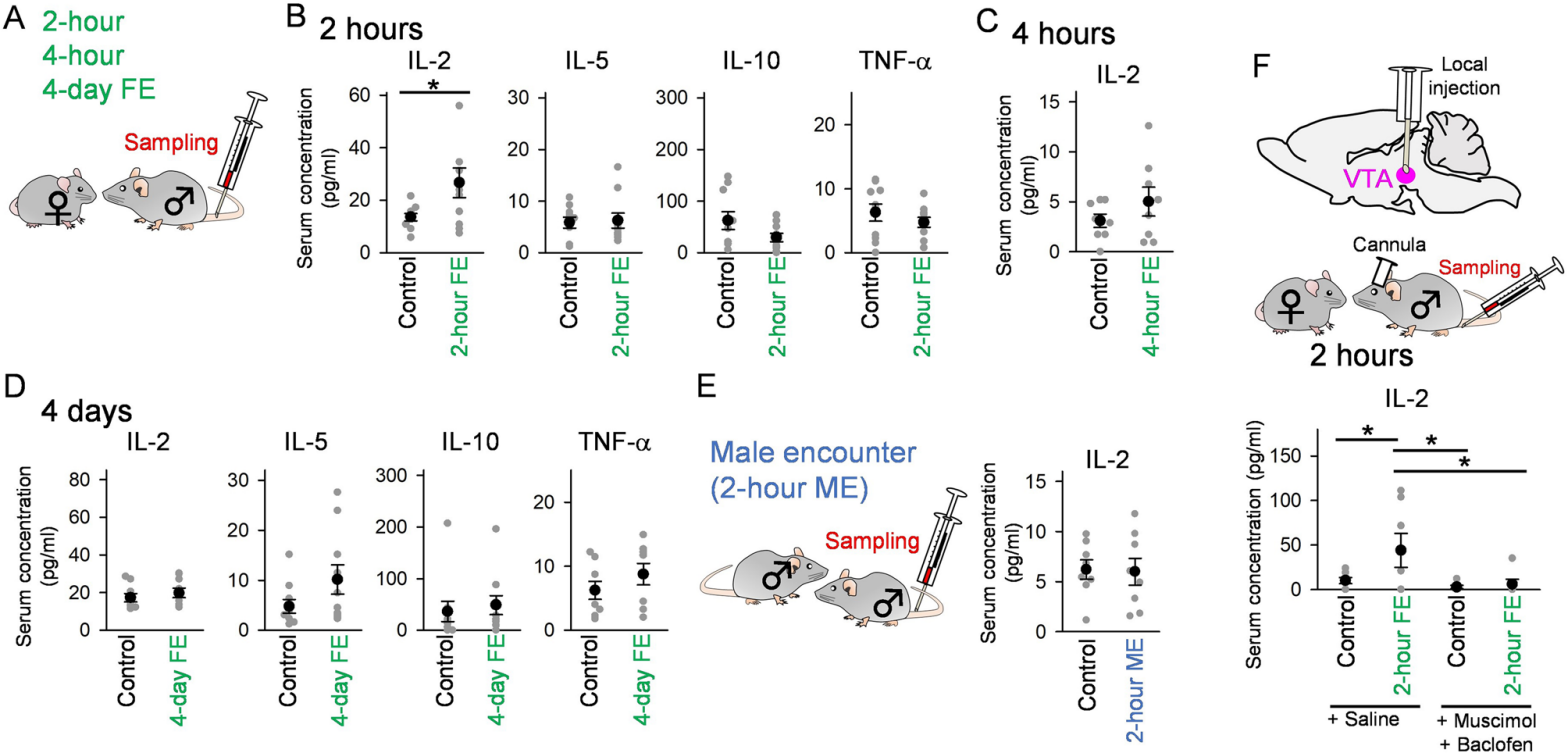
Female encounters increase serum cytokine levels, which are abolished by VTA inactivation. (**A**) A male mouse was subjected to a FE for 2 h, 4 h or 4 days, and a blood sample was collected. (**B**) Serum concentrations of four cytokines after a 2-h FE (Control, *n* = 10 mice; 2-h FE, *n* = 10 mice). Each thin dot represents one mouse. *P < 0.05. (**C**) Same as B but for a 4-h FE (Control, *n* = 8 mice; 4-h FE, *n* = 8 mice). (**D**) Same as B but for a 4-day FE (*n* = 7–10 mice). (**E**) (Left) A male mouse freely interacted with the other male mouse for 2 h, termed a male encounter (ME), and blood sample was collected. (Right) Serum IL-2 concentrations after a 2-h ME (Control, *n* = 8 mice; ME, *n* = 8 mice). (**F**) (Top) To inhibit neuronal activity in the VTA, muscimol and baclofen were locally injected into the VTA of male mice subjected to a 2-h FE. (Bottom) Changes in serum IL-2 concentrations after inhibition of the VTA (*n* = 6–7 mice). *P < 0.05.

The observations of reward circuit activation by a FE suggest that this experience may induce peripheral immune responses, similar to VTA photostimulation. We addressed this idea by collecting a blood sample immediately after the 2-h FE (Fig. 3A). After the 2-h FE, the serum IL-2 concentration was significantly increased compared with control conditions without FE (Fig. 3B; *t*_19_ = 2.11, *P* = 0.048, Student’s *t*-test), while serum IL-5, IL-10, and TNF-α concentrations were unchanged (IL-5: *t*_19_ = 0.22, *P* = 0.83; IL-10: *t*_19_ = 1.70, *P* = 0.11; TNF-α: *t*_19_ = 0.98, *P* = 0.34, Student’s *t*-test). The same test was performed by extending the period of FE to 4 h but no significant change in IL-2 was observed in this condition (Fig. 3C; *t*_15_ = 1.19, *P* = 0.25). The increase in IL-2 was no longer observed when mice were continuously exposed to FE for 4 days (Fig. 3D; IL-2: *t*_17_ = 0.76, *P* = 0.46; IL-5: *t*_19_ = 1.62, *P* = 0.12; IL-10: *t*_19_ = 0.47, *P* = 0.65; TNF-α: *t*_13_ = 0.90, *P* = 0.35, Student’s *t*-test). These results demonstrate that the experience of a FE at a certain period induces a subset of serum cytokine levels, similar to the results from VTA photoactivation. To reveal whether the increase in serum IL-2 concentrations was induced by the novelty of the other mouse, irrespective of animal’s sex, we tested a male encounter (ME) paradigm in which a male mouse interacted with the other male mouse for 2 h (Fig. 3E). This paradigm did not change serum IL-2 concentrations (Fig. 3E; *t*_15_ = 0.13, *P* = 0.90, Student’s *t*-test). Taken together, these results suggest that the increase in serum IL-2 concentrations was specific to female mice, rather than male mice or the novelty of the other mice alone. Finally, we tested whether the female encounter-induced effect was mediated by the VTA. To inhibit VTA neuronal activity, a mixture of muscimol, a GABA_A_ receptor agonist, and baclofen, a GABA_B_ receptor agonist, was locally injected into the VTA (Fig. 3F). Drug injection resulted in no significant increases in their serum IL-2 levels after a 2-h FE (*P* > 0.05, Tukey’s test after two-way ANOVA). On the other hand, serum IL-2 levels were significantly increased by a FE in mice not injected the drugs (effect of FE : *F* (1, 21) = 4.71, *P* = 0.042, two-way ANOVA; Saline Control vs Saline FE : *P* = 0.047, Tukey’s test). Furthermore, in the case the FE groups were compared with each other, the injection of the drugs significantly decreased the serum IL-2 levels (effect of the drugs : *F* (1, 21) = 5.89, *P* = 0.024, two-way ANOVA; Saline FE vs Drug FE : *P* = 0.033, Tukey’s test).These results suggest that VTA neuronal activity is necessary for FE-induced elevation of serum cytokine levels.

## Discussion

We designed our study to examine whether reward system activation can impact immunity. We demonstrated that phasic, rather than tonic, optogenetic stimulation of VTA dopaminergic neurons effectively increased peripheral cytokine levels. In addition, the experience of a FE, a natural condition, was sufficient to trigger a cytokine increase through the activation of the VTA in male mice. These results suggest that the immune system responds to rewarding stimuli depending on phasic VTA dopaminergic activity.

Recent studies have begun to unveil the role of brain activity related to reward-related behavior and experiences in the induction of peripheral immune responses. For instance, human studies have demonstrated that mirthful laughter is a useful cognitive intervention that can activate NK cells^30^, and positive future expectations driven by placebo medication can ameliorate patients’ pathological conditions^31,32^. In contrast, chronic stress and depression are risk factors for immune dysfunctions, leading to increased incidence of infections and a wide range of immune-related diseases^33–36^, while mild stress (e.g., acrophobic stress) may be rather effective in enhancing the antibody response^37^. Although evidence of emotion-related immune reactions has accumulated, the detailed physiological mechanisms and dynamics have not been fully elucidated. Using animal models, a pioneering study demonstrated that the chemogenetic activation of VTA neurons by the DREADD system boosts antibacterial and antitumor cytotoxic activity^13,14^. In addition, activation of corticotropin-releasing hormone-containing neurons in the paraventricular nucleus of the hypothalamus by the DREADD system facilitates splenic plasma cell formation^37^. These psychopharmacological methods inevitably activate targeted brain neurons without temporal specificity, making it impossible to identify detailed physiological activity patterns on the millisecond time scale, a time window that is crucial for information processing by neurons. Optogenetic tools allow for precisely timed control of specific types of neurons and are expected to be useful methods to test the regulation of immunity by the central nervous system^19^. Consistent with the importance of their phasic firing in encoding reward signals, we demonstrated that phasic, rather than tonic, optogenetic activation of VTA dopaminergic neurons could trigger immune responses. These results support causal relationships between specific brain activity (e.g. phasic activity) of VTA dopaminergic neurons and the ensuing changes in immune functions. We note that, as midbrain dopamine neurons have been shown to co-release glutamate or GABA^23^, all of our results presented in this study may be mediated by not only dopaminergic signals but also the coordination with glutamatergic or GABAergic signaling from VTA dopaminergic neurons. In addition, our optogenetic stimulation lasted for at least 2 h, which is considerably longer than that used in previous studies (up to 1 h)^16,20^. It remains to be determined whether dopaminergic neuronal activation in such shorter periods could mimic the same effects.

The main pathways for immunoenhancement effects by the brain are the HPA axis and the peripheral nervous system^1,2,23^. In particular, the peripheral immune system is mainly innervated by the sympathetic nervous system^38^, and the release of several cytokines, including IL-2, which is a main cytokine that was focused on in this study, from immune cells is mainly controlled via the activation of β2-adrenergic receptors^39,40^. Consistently, removal of the sympathetic nerve abolishes VTA-induced immune effects^13,14^. Taken together, the VTA-induced immune effects observed in this study are likely mediated by the sympathetic nervous system.

We showed that immune effects could be induced by a FE, a natural condition that elicits c-Fos expression in the VTA and NAc shell, which is consistent with previous findings^10,21,41^. In addition, we showed that pharmacological inhibition of VTA neuronal activity abolished pronounced increases in serum IL-2 levels by a FE, suggesting the necessity of VTA neurons in immune responses. However, we note that this pharmacological inhibition affects all neuron types in the VTA without the specificity of dopaminergic neurons. To more precisely determine whether the FE-induced immune responses are mediated by VTA dopaminergic neurons, experiments with optogenetic or chemogenetic tools that can selectively inhibit specific neuron types in the VTA are required.

As with other natural conditions, housing of mice in an enriched environment has been shown to be effective in elevating VTA dopaminergic neuronal activity^42^, enhancing NK cell activity^43^, and even suppressing pancreatic cancer^44^. These scattered pieces of evidence all support the idea that a positive mental state boosts immunoreactivity, offering a new direction for therapy without chemical or invasive interventions, such as drugs and surgeries, in many immune-related disorders. Further studies are needed to generate a comprehensive map of the beneficial dialogue between the immune and reward systems.

## Methods

### Approvals

Animal experiments were performed with the approval of the Animal Experiment Ethics Committee at The University of Tokyo (approval number: P29-14) and according to the ARRIVE guidelines and the University of Tokyo guidelines for the care and use of laboratory animals. These experimental protocols were carried out in accordance with the Fundamental Guidelines for Proper Conduct of Animal Experiment and Related Activities in Academic Research Institutions (Ministry of Education, Culture, Sports, Science and Technology, Notice No. 71 of 2006), the Standards for Breeding and Housing of and Pain Alleviation for Experimental Animals (Ministry of the Environment, Notice No. 88 of 2006) and the Guidelines on the Method of Animal Disposal (Prime Minister's Office, Notice No. 40 of 1995). All efforts were made to minimize the animals’ suffering.

### Subjects

Male C57BL/6 J wild-type mice (8–14 weeks old) with preoperative weights of 20–30 g were used in this study. All mice were purchased from SLC (Shizuoka, Japan). In addition, DAT-IRES-Cre + /− mice (Jackson Laboratories, B6;SJL-Slc6a3tm1.1(cre)Bkmn/J, stock number 006660) were crossed with Ai:32 (RCL-ChR2(H134R)/EYFP) mice (Jackson Laboratories, B6;129S-Gt(ROSA)26Sortm32(CAG-COP4*H134R/EYFP)Hze/J, stock number 012569) to yield DAT-Cre × RCL-ChR2-eYFP mice. The transgenic mice were used when they were 2–6 months old with preoperative weights of 20–35 g. The animals were housed and maintained on a 12-h light/12-h dark schedule with the lights off at 7:00 PM and received surgery for the implantation of an electrode assembly.

### Surgery

For all surgeries, the mice were anesthetized with isoflurane gas (1–2%) in air and then fixed in a stereotaxic instrument with two ear bars and a nose clamp. An incision was made from the area between the eyes to the back of the head, and circular craniotomies with a diameter of ~ 1.0 mm were made using a high-speed drill at the indicated coordinates. For optogenetic experiments, an optical fiber (core diameter = 200 µm) was implanted into the VTA (3.1 mm posterior and 0.3 mm lateral to the bregma) at a depth of 4.5 mm. For local field potential (LFP) recordings, an electrode assembly created by a 3D printer^45–47^ that consisted of 3 immobile electrodes was implanted into the VTA and the medial prefrontal cortex (mPFC) (2.00 mm anterior and 0.50 mm lateral to the bregma at a depth of 1.40 mm). The electrodes were constructed from 17-μm-wide polyimide-coated platinum-iridium (90/10%) wires (California Fine Wire), and the electrode tips were plated with platinum to lower the electrode impedances to 200–250 kΩ. For the cerebellum, stainless steel screws were implanted in the skull and attached to the brain surface to serve as ground/reference electrodes. For local drug injection, a guide cannula (8 mm length, inner diameter = 0.34 mm and outer diameter = 0.5 mm, AG-8, Eicom, Japan)^48^ was implanted into the VTA at the same coordinates. To prevent drying of the implanted region, a dummy cannula was inserted through the guide cannula, which were both covered with a cap. Finally, the device was secured to the skull using stainless steel screws and dental cement. After all surgical procedures were completed, anesthesia was discontinued, and the animals were allowed to awaken spontaneously. Following surgery, each animal was housed in a transparent Plexiglas cage with free access to water and food for more than 1 week.

### Optogenetics

The mice underwent one of the following photostimulation protocols in a plastic cage: (1) tonic stimulation with single pulses (~ 30 mV, 15 ms) continuously applied at 2.5 Hz, or (2) phasic stimulation with 25 pulses of 15-ms blue light delivery (472 nm, ~ 30 mW output from fiber) at 50 Hz applied with a periodicity of 10 s.

### Electrophysiology

Each mouse was connected to the recording equipment via Cereplex M (Blackrock), a digitally programmable amplifier, close to the animal’s head. The headstage output was conducted via a lightweight multiwire tether and a commutator to the Cereplex Direct recording system (Blackrock), a data acquisition system. LFP recordings were sampled at 2 kHz and filtered between 0.1 and 500 Hz.

### Drug injection

For drug injection into the VTA, the dummy cannula was removed from the guide tube and replaced by a plastic injection cannula with a diameter of 0.34 mm so that the tip of the injection cannula reached above the VTA. The other side of the injection cannula was connected by polyethylene tubing to a 5-μl syringe mounted in an infusion pump (KDS LEGATO101, Muromachi, Japan). Through the injection cannula, a mixture of 1 mM muscimol and 50 μM baclofen dissolved in saline (pH 7.4) was infused into the VTA at a rate of 0.1 μl/min for 2 min. After the infusion was complete, the injection cannula was left in place for 1 min, and the mice were placed back in the rest box for 1 h. Then, the dummy cannula was again inserted into the guide tube. During the drug injection procedure, the animals did not show any sign of stress or discomfort.

### Blood sampling and measurement of serum cytokine levels by ELISA

Blood samples were collected from the right ventricle or caudal vein under anesthesia with isoflurane (1–2%) and stored at 4 °C for 6 h. The blood samples were centrifuged at 1000×g for 20 min to obtain the serum, which was preserved at −80 °C until analysis. The concentrations of serum cytokines were measured by using BioPlex (Bio-Rad) or ELISA kits (Proteintech) as shown in Figs. 1D, E, 3B–F, respectively.

### Immunochemistry

The mice received an overdose of urethane and were perfused intracardially with cold 4% paraformaldehyde (PFA) in 25 mM PBS and decapitated. The brains were placed in 30% sucrose until equilibrated and coronally sectioned at a thickness of 50 µm. To obtain brain slices, the fixed samples were rinsed with PBS and then permeabilized in 100 mM PBS with 0.3% Triton X-100 and 5% serum bovine albumin (Nacalai Tesque, Kyoto, Japan) at room temperature for 60 min. The slices were then incubated with a primary mouse anti-tyrosine hydroxylase (TH) antibody (1:1000, EMD Millipore, USA), primary chicken anti-GFP antibody (1:1000, Abcam, UK), or primary rabbit anti-c-Fos antibody (1:500 for VTA, 1:1000 for the other brain regions, Synaptic Systems, Goettingen, Germany) in 100 mM PBS with 0.3% Triton X-100 and 5% serum bovine albumin (Nacalai Tesque, Kyoto, Japan) for one overnight period at 4 °C. After rinsing with PBS, they were then labeled with a secondary anti-rabbit IgG antibody Alexa 405 (1:500; Thermo Fisher Scientific, Tokyo, Japan), anti-rabbit IgG antibody Alexa 488 (1:500; Thermo Fisher Scientific, Tokyo, Japan), secondary anti-chicken IgG Alexa 488 (1:500; Thermo Fisher Scientific), or secondary anti-mouse IgG Alexa 647 (1:500; Thermo Fisher Scientific) in 100 mM PBS with 0.3% Triton X-100 for 90 min. The samples were mounted using vectashield with DAPI (Funakoshi Co., Ltd, Tokyo, Japan) or without DAPI (Falma, Tokyo, Japan). Images were acquired at a Z-depth interval of 2 μm using a confocal laser-scanning microscope (BX51-FL; Olympus, Tokyo, Japan) with a water immersion objective lens (× 10, 0.4 NA; × 20, 0.75 NA).

### Histological analysis to confirm electrode placement

For the reconstruction of the electrode tracks, the electrodes were not withdrawn from the brain until 8–12 h after perfusion. After dissection, the brains were fixed overnight in 4% PFA and then equilibrated with a sequence of 20% sucrose followed by 30% sucrose in PBS. Frozen coronal Sects. (50 μm) were cut using a microtome, and serial sections were mounted and processed for cresyl violet staining. For cresyl violet staining, the slices were rinsed in water, counterstained with cresyl violet, and coverslipped with Permount. The positions of all tetrodes were confirmed by identifying the corresponding electrode tracks in the histological tissue. Recordings were included in the data analysis if the tetrode’s deepest position was in the targeted region.

### Statistical analysis

All data are presented as the mean ± standard error of the mean (SEM). Comparisons of two-sample data were analyzed using Student’s *t*-test or paired *t*-test. Comparisons of three or more data points were performed using Tukey’s test after one-way or two-way analysis of variance (ANOVA).

## Data Availability

The data that support the findings of this study are available from the corresponding authors upon reasonable request.
